# Alveolar Bone Segmentation Methods in Assessing the Effectiveness of Periodontal Defect Regeneration Through Machine Learning of CBCT Data: A Systematic Review

**DOI:** 10.1155/ijbi/9065572

**Published:** 2025-12-21

**Authors:** Mahmud Mohammed, Tulio Fernandez-Medina, Manjunath Rajashekhar, Stephanie Baker, Ernest Jennings

**Affiliations:** ^1^ College of Medicine and Dentistry, James Cook University, Cairns, Queensland, Australia, jcu.edu.au; ^2^ Delta Medical College, Dhaka, Bangladesh; ^3^ School of Dentistry, The University of Queensland, Brisbane, Australia, uq.edu.au; ^4^ College of Science and Engineering, James Cook University, Cairns, Queensland, Australia, jcu.edu.au

**Keywords:** alveolar bone segmentation, artificial intelligence, CBCT, deep learning, digital, machine learning, segmentation

## Abstract

**Objectives:**

To evaluate various segmentation methods used for cone‐beam computed tomography (CBCT) images of alveolar bone, assessing their effectiveness and potential benefits in digital workflows for periodontal defect regeneration.

**Data:**

This review adheres to the PRISMA‐ScR (Preferred Reporting Items for Systematic Reviews and Meta‐Analyses Extension for Scoping Reviews) Checklist.

**Source:**

A comprehensive literature search was conducted from May 2024 to June 2025 using MeSH terms on PubMed, Scopus, Web of Science, and Medline databases, with publication date restricted to 5 years. The PRISMA guidelines were followed to ensure a systematic review process, and the review protocol was registered with Prospero. The QUADAS‐2 checklist was used to evaluate the risk of bias in the included studies.

**Study Selection:**

The initial search yielded 834 articles, which were systematically filtered down to 23 eligible studies. Deep learning methods, particularly U‐Net, were the most frequently employed segmentation techniques. Four studies utilized semi‐automated methods, while the remaining studies relied on manual or other segmentation methods. The Dice similarity (DC) index, ranging from 76% to 98%, was the primary metric used to assess segmentation performance.

**Conclusions:**

Significant differences were observed between the segmentation of healthy and defective alveolar bone, underscoring the need to enhance deep learning–based methods. Accurate segmentation of periodontal defects in DICOM images is a crucial first step in the scaffold workflow, as it enables precise assessment of defect morphology and volume. This information directly informs scaffold design, ensuring that the scaffold geometry is tailored to the patient‐specific defect.

**Prospero Registration:**

CRD42024590957.

## 1. Introduction

Cone‐beam computed tomography (CBCT) technology has been increasingly incorporated into radiology and has found extensive use in various dental disciplines, including implant design, periodontology, endodontics, orthodontics, and maxillofacial surgery [1–3]. In periodontology, CBCT plays a crucial role in assessing periodontal bone defects, which are areas of bone loss surrounding teeth typically caused by periodontal disease. This condition compromises the supporting tissues due to infection and inflammation. These defects can vary, including vertical (intra‐bony) defects that occur along the tooth root, and horizontal defects, where bone loss is more uniform [4]. Treatments commonly include scaling and root planning, bone grafting, guided tissue regeneration, and sometimes the use of specific proteins to promote bone and tissue regeneration [5].

In recent years, tissue engineering strategies with the potential to induce three‐dimensional periodontal tissue regeneration have been explored. The combination of biocompatible scaffolds, bioactive molecules, and cells aims to replicate structural tissue organization and induce de novo periodontal tissue regeneration [6]. To design and manufacture patient‐specific scaffolds, Digital Imaging and Communications in Medicine (DICOM) images obtained from CBCT scans are essential components of the workflow [7]. The exact dimensions and morphology of periodontal defects are commonly visible on CBCT images, as they can provide cross‐sectional views in various orientations (coronal, sagittal, and axial planes). Once images are acquired, the process of image segmentation takes place, playing an essential role in the quality and reliability of the resulting images.

Segmentation is the process of isolating specific structures or areas of interest from surrounding tissue within CBCT scans [8]. In periodontal contexts, this means distinguishing structures such as teeth, bone, and soft tissue. This technique enables the creation of accurate 3D models that are valuable for diagnosis, treatment planning, and the design of customized scaffolds in tissue engineering [9].

Typically, segmentation is done with specialized software that processes the DICOM data from CBCT scans, identifying boundaries between different tissue types. This allows clinicians to gain a clearer understanding of the dimensions, shape, and position of defects or targeted areas, facilitating the development of precise, patient‐specific treatment strategies [10, 11]. This can be performed by using a variety of methods, including traditional methods, deep learning, and hybrid approaches [12]. Traditional techniques, such as thresholding, region growing, and watershed segmentation, rely on simple image processing methods and are susceptible to noise and variations in image quality [13]. Deep learning techniques, such as convolutional neural networks (CNNs), U‐Net, and 3D fully convolutional networks (FCNs), provide more advanced and accurate segmentation capabilities by learning intricate patterns from extensive datasets [14]. Hybrid approaches combine the strengths of traditional and deep learning methods. These approaches typically involve using deep learning for initial segmentation, followed by traditional methods for refinement, resulting in enhanced overall segmentation performance. [15]. Segmentation quality is commonly assessed using the Dice coefficient (DC), a statistical measure of the similarity between two sets of data, such as those generated by automated and manual segmentation algorithms in CBCT image analysis. A higher DC indicates better agreement between the two segmentations, with a perfect match resulting in a score of 1.0. This metric is widely used in medical image analysis to evaluate the performance of segmentation algorithms [16].

**Figure 1:**
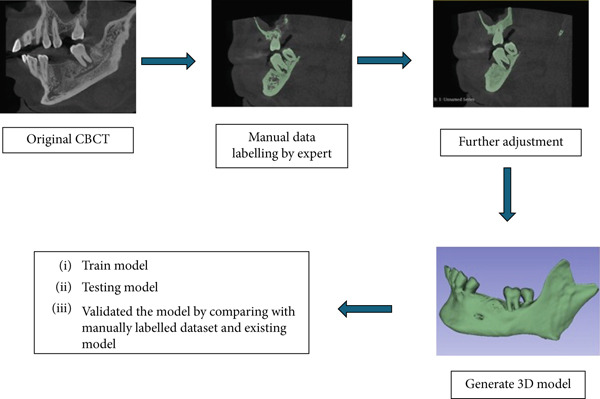
Stage‐by‐stage image illustrations showing the segmentation process.

Accurate segmentation of the affected alveolar bone is crucial for the fabrication of precise 3D‐printed periodontal scaffolds. The use of fundamental thresholding methods may result in 3D dental models with increased noise and reduced accuracy (Figure 1). In addition to basic thresholding, other automated techniques, such as the level set method, have been employed for the segmentation of CBCT images in dental applications including CNNs [17, 18], and the use of morphological operators in conjunction with the watershed algorithm [19].

However, technical difficulties related to DICOM image segmentation have been previously reported [20]. Due to the similarities in intensities, the narrow spacing between adjacent teeth, and the complex anatomical structures of the periodontal apparatus, segmenting these structures remains challenging [21]. Deep learning algorithms have demonstrated potential for segmenting teeth and alveolar bone from CBCT data for 3D reconstruction [22]. By employing neural networks, segmentation of the alveolar bone and teeth is possible, even in the presence of significant metal artifact interference. While most studies have relied on simple thresholding methods for generating 3D models, the application of specific semi‐automated techniques for segmenting the alveolar bone, particularly using commercially available segmentation software, remains limited.

### 1.1. Clinical Motivation

Accurate assessment of alveolar bone on CBCT is essential for diagnosing and managing periodontal disease and designing patient‐specific 3D printed periodontal scaffolds. However, manual segmentation is labor‐intensive, time‐consuming, and subject to operator variability, limiting its feasibility in routine clinical practice. Deep learning approaches can be an alternative to reduce these limitations, and their clinical application can generate more accurate and patient‐specific scaffolds for treating periodontal bone loss.

### 1.2. Research Aim

This study aims to systematically evaluate the available methods for segmentation of alveolar bone from CBCT images to identify the most suitable method for generating accurate 3D models for periodontal defect regeneration, thereby advancing the field of digital dentistry and enabling personalized treatment planning.

## 2. Materials and Methods

This systematic review follows the guidelines of the Preferred Reporting of Systematic Review (PRISMA) statement [23]. The review protocol was registered with the international database of prospectively registered systematic reviews in health and social care‐Prospero (CRDxxxxxxxx7).

*PICO Question (Population, Intervention, Comparison, Outcomes)*

“How do different DICOM segmentation methods for CBCT images of alveolar bone influence the accuracy and efficiency of digital workflows, ultimately impacting the precision of 3D models used in periodontal defect regeneration?”

The PICO question was defined as follows:

(P) 3D data with alveolar bone

(I) Segmentation of alveolar bone following sectioning

(C) Methods for the segmentation of alveolar bone in 3D data

(O) Segmented alveolar bone

### 2.1. Searching Strategies

The literature search was conducted using the following electronic bibliographic databases by two different authors (MM and EJ): Scopus, PubMed, Web of Science, and Medline databases (searched from May 1, 2024, up to June 12, 2025). Grey literature was also searched using the Open Grey database (http://www.opengrey.eu/) with the same search strategy as the other databases. To ensure comprehensive coverage, reference lists of all included studies were hand‐searched for relevant articles not identified in the electronic searches.

Comprehensive search strategies including thesaurus terms (MeSH terms) were used (Table 1) to increase the accuracy and completeness of the search results: (segmentation OR deep learning OR machine learning OR neural network OR artificial intelligence) AND (CBCT OR “cone beam” OR “cone‐beam” OR “volum∗ ct” OR “volume computed tomography” OR “3d imag∗” OR “three dimensional imag∗”)) AND (alveolar OR “bone loss” OR “periodontal resorption∗”). There were no language restrictions, and the publication period was restricted to less than 5 years. The searches were re‐run just before the final analyses, and further studies were retrieved for inclusion.

**Table 1:** Keyword searching.

**Database**	**Keywords**	**Studies (*n*)**
PubMed	((segmentation OR deep learning) AND (CBCT OR “cone beam” OR “cone‐beam” OR “volum∗ ct” OR “volume computed tomography” OR “3d imag∗” OR “three dimensional imag∗”)) AND (alveolar OR “bone loss” OR “periodontal resorption∗”)	165

Medline	Image Interpretation, Computer‐Assisted/OR Tomography, X‐ray Computed/OR Image Processing, Computer‐Assisted/OR Neural Networks, Computer/OR segmentation.mp. OR Deep Learning OR Imaging, Three‐Dimensional.mp.OR Imaging, Three‐Dimensional AND deep learning.mp. OR Deep Learning/OR Algorithms OR segmentation AND Periodontitis/OR Alveolar Bone Loss OR alveolar bone.mp.	52

Scopus	(TITLE‐ABS‐KEY (cbct OR “cone beam” OR “cone‐beam” OR “volum∗ ct” OR “volume computed tomography” OR “3d imag∗” OR “three dimensional imag∗”)) AND (TITLE‐ABS‐KEY (alveolar OR “bone loss” OR “periodontal resorption∗”)) AND (TITLE‐ABS‐KEY (segment∗ OR “deep learning”))	465

Web of Science	Segment∗ OR “deep learning”AND alveolar OR “bone loss” OR “periodontal resorption∗” AND CBCT OR “cone beam” OR “cone‐beam” OR “volum∗ ct” OR “volume computed tomography” OR “3d imag∗” OR “three dimensional imag	152

### 2.2. Searching Criteria

The screening process was aligned with the PRISMA guidelines. We transferred all the search items from different search engines into EndNote (version 21). We eliminated all the duplicate manuscripts using EndNote (version 21). Further manual search was carried out to remove any remaining duplicate materials. The study selection was guided by the PICO questions.

### 2.3. Observational and Considerable Parameters/Data Extraction

To ensure comprehensive coverage, the reference lists of included studies were manually searched for additional relevant articles. All records were screened against predetermined inclusion and exclusion criteria, and full‐text articles were assessed for eligibility and qualitative synthesis. Data were extracted from each study, including the data acquisition device, segmentation method, sample size, effect of segmentation on results, and general outcomes. An initial screening of titles and abstracts was conducted independently by two reviewers (MM and EJ). Inter‐observer reliability was assessed using Cohen’s kappa coefficient, with a threshold of ≥ 0.8 indicating substantial agreement [24]. In all stages of the selection process, discrepancies between reviewers were discussed until consensus was reached.

The primary outcome measure was the accuracy of the segmentation method used to extract the alveolar bone. Secondary outcomes included comparisons between segmentation methods and assessments of the effectiveness of the interventions.

### 2.4. Risk of Bias Assessment

Due to the significant heterogeneity in outcome definitions across studies, a statistical assessment of overall bias risk for case–control and cohort studies was not appropriate. However, the Quality Assessment Tool for Diagnostic Accuracy Studies 2 (QUADAS‐2) checklist was employed to evaluate the risk of bias in diagnostic accuracy studies [25].

Two independent reviewers (MM and EJ) assessed the risk of bias for each selected study using the QUADAS‐2 tool. This tool evaluates four key domains: index tests, reference tests, patient selection, and flow diagram. Each domain was assigned a risk of bias rating of high, low, or unclear. If one or more key domains were rated as high risk, the study was classified as high risk of bias. If two or more key domains were rated as unclear, the study was categorized as unclear risk of bias.

## 3. Results

### 3.1. Study Selection

**Figure 2:**
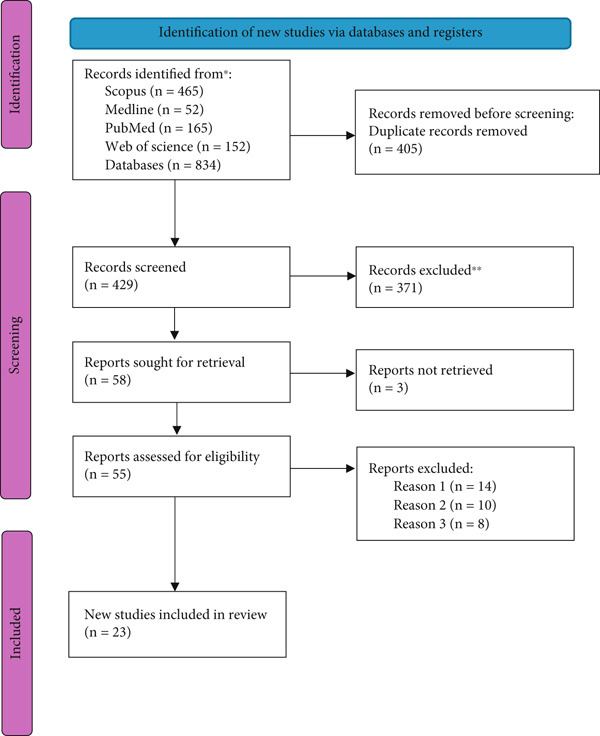
PRISMA flow diagram for the systematic reviews, which included searches of databases.

A comprehensive literature search was conducted across multiple databases (Table 1). Among the 834 identified studies, 405 were duplicates and excluded. After screening titles and abstracts, 55 studies remained. Following a full‐text review, 32 studies were excluded due to the exclusion criteria. Ultimately, 23 studies were selected for inclusion in this review (Figure 2).

### 3.2. Study Characteristics

**Figure 3:**
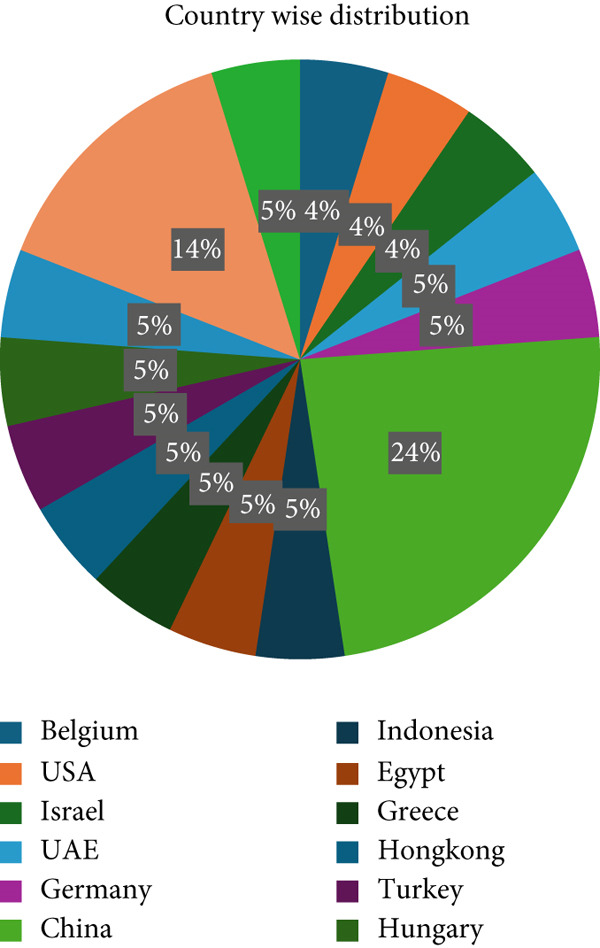
Country‐wise distribution of the studies.

Out of the 23 selected studies, five were conducted in China and three in the Netherlands. One study each was performed in Hong Kong, Germany, Greece, Belgium, Switzerland, Hungary, United States, UAE, Egypt, Turkey, Indonesia, Italy, and Israel (Figure 3). Most of the papers that met the inclusion criteria originated from Europe (10 out of 23). This occurrence is coincidental, and there is no bias present in the search methodology.

Among the 23 included studies, 22 included the CBCT data for alveolar bone segmentation in human samples [20, 21, 26–44]. The remaining one study included micro‐CT data for alveolar bone segmentation in a mouse model. [42]. Six studies segmented the defective alveolar bone in addition to the healthy alveolar bone [30–32, 42]. Only four studies segmented the alveolar bone in periodontal patients [32, 42–44]. One of these studies used a mouse model and micro‐CT for segmenting periodontally diseased alveolar bone [42].

### 3.3. Methods of Segmentation

#### 3.3.1. Deep Learning Techniques

Out of 23 studies, 14 (Table 2) used the deep learning segmentation technique to extract the alveolar bone from CBCT data [21, 27–30, 36, 38–41]. Most of the studies revealed a high DC ranging from 76% to 98%. The number of samples ranged from 36 to 389 patients for most of the studies, with one study having a high sample size of 4215 patients. Among these studies, only one study used a micro‐CT scanner for data acquisition. The most popular deep learning algorithm was U‐Net segmentation. Out of 14 studies, 10 used the U‐Net technique for segmenting the alveolar bone. Only four studies used other techniques than U‐Net, such as V‐Net, Instance segmentation, multi‐structure segmentation, and MS‐D network for segmenting alveolar bone from CBCT images [21, 41, 45, 46]. Only four studies used deep learning architecture for segmenting defective alveolar bone. Only two studies used deep learning architecture for segmenting periodontally defective alveolar bone in human samples.

**Table 2:** Description of the studies (deep learning segmentation).

**Authors, year of publication**	**Country**	**Aim**	**Alveolar bone type**	**DSC**	**IoU**	**Precision**	**Sensitivity**	**Samples**	**Dataset for validation test** **Training:validation:testing**	**Image modality**	**Segmentation methods/tools**
Fontenele, Gerhardt et al., 2023 [29]	Belgium	Maxillary alveolar bone segmentation.	Healthy	95.4 ± 0.6	91.2 ± 1.2	93.0 ± 1.5		141 CBCT scan	99:12:30	CBCT	3D U‐Net

Xi, Ali et al., 2024 [42]	USA	Analysis of *μ*CT data to measure changes in mouse alveolar bone during ligature‐induced periodontitis mouse model.	Defected	98%				2763 slices (12 mice)	NA	Micro CT	U‐Net

Yeshua, Ladyzhensky et al., 2023 [30]	Israel	3D segmentation of various maxillofacial bone lesions in CBCT scans.	Defected	83.5%		98.9%	95.9%	82 patients	50:10:22	CBCT	U‐Net (Mask‐RCNN)

Moufti, Trabulsi et al., 2023 [39]	UAE	Segmentation of the edentulous spans on CBCT.	Healthy	78%				43 CBCT images	43:10:33	CBCT	U‐Net

Liu, Xie et al., 2024 [36]	China	To segment alveolar bone, tooth. IAN canal and maxillary sinus.	Healthy	94.3 ± 1.0	±1.1 79.5 (max)			NA	NA	CBCT	U‐Net (Skeletal segmentation network)

Su, Jia et al., 2024 [41]	China	Segmentation of Tooth PDL and alveolar bone.	Healthy	90.8 ± 3.2	83.6 ± 2.0			389 patients and 1734 axial CBCT images	80:20	CBCT	Instance segmentation (Mask R‐CNN)

Widiasri, Suciati et al., 2023 [38]	Indonesia	Segment Alveolar Bone and Mandibular Canal regions.	Healthy		98%			563 2D grayscale cone‐beam computed	NA	CBCT	U‐Net

Abdo, Mohamed et al., 2022 [27]	Egypt	Use deep learning models to segment jaws into upper and lower jaws.	Healthy	91%				117	4:1	CBCT	U‐Net (Attention Unet+ Normalization + Mask)

Chen, Chen et al., 2023 [28]	China	Dental CBCT image segmentation.	Healthy	87.12%	78.9%			200	6:2:2	CBCT	U‐Net. (CNN‐Transformer Architecture)

Cui, Fang et al., 2022 [21]	Hongkong	Use deep learning models to segment the tooth and alveolar bone.	Healthy	94.1% (maxilla) 94.8% (mandible)			93.5% (maxilla) 94.2% (mandible	4215 (4938 CBCT scans of 4215 patients).	7:7:3	CBCT	CNN (2 stages: V‐Net + hierarchical morphology‐guided network).

Kurt‐Bayrakdar et al. 2025[44]	Turkey	Automatically detect tooth presence, tooth numbering, and types of periodontal bone defects from cone‐beam CT (CBCT) images using a segmentation method with an advanced artificial intelligence (AI) algorithm.	Defected	76%				502	251:251	CBCT	U‐Net (nnU‐Net v2)

Palkovics et al. 2025[43]	Hungary	Evaluated the performance of a multi‐stage Segmentation Residual Network (SegResNet)‐ based deep learning (DL) model for the automatic segmentation of cone‐beam computed tomography (CBCT) images of patients with stage III and IV periodontitis	Defected	96.5 ± 0.97%	93.4 ± 1.8%			70	57:13:10	CBCT	3D U‐Net (SegResNet)

Wodzinski and Müller 2025[45]	Switzerland	Automatic multi‐structure segmentation in cone‐beam computed tomography	Healthy	87.8% (mandible) 73.8% (maxilla0				480	417:63:42	CBCT	Multi‐structure segmentation (ToothFairy2)/

Minnema et al. 2019 [46]	Netherland	In order to attain anatomical models, surgical guides and implants for computer‐assisted surgery, accurate segmentation of bony structures in cone‐beam computed tomography (CBCT	Healthy	87 ± 6%				20	18:2	CBCT	MS‐D network

Abbreviations: AI, artificial intelligence; CBCT, cone‐beam computed tomography; CCN, convolutional neural network; IAN, inferior alveolar nerve; MCI, morphological contour interpolation; PDL, periodontal ligament; RCNN, rapid convolutional neural network.

#### 3.3.2. Manual and Semiautomated Segmenting

Four studies (Table 3) performed semiautomated methods for segmenting the alveolar bone from CBCT data [31, 32, 34, 35]. The DC of the semi‐automated segmentation method was only mentioned by one study, which achieved 97% [29], while other studies used different evaluation metrics to check the accuracy of their methods. The sample size ranged from 11 to 67. Three studies (Table 4) applied manual segmentation or threshold‐based segmentation techniques for segmenting the alveolar bone from CBCT images [20, 33, 40]. Only one study reported [20] a DC of 95.64% for a semi‐automated segmentation method. Other studies used different evaluation metrics to assess accuracy. Sample sizes ranged from 6 to 9. Two studies (Table 5) used different approaches, such as morphological contouring for the segmentation of alveolar bone [26, 37]. The DC was mentioned by only one study (84%). The sample sizes of the studies were 106 and 20 datasets, respectively.

**Table 3:** Description of the studies (semiautomated segmentation).

**Authors, year of publication**	**Country**	**Aim**	**Dice coefficient score (DCS)**	**Samples**	**Software**	**Dataset for validation test** **Training:validation:testing**	**Image modality**	**Segmentation methods/tools**
Zirk, Buller et al., 2019 [31]	Germany	Segmentation of medication related osteonecrosis of the jaw lesion.	NA	67 patients	ITK SNAP	NA	CBCT	Semiautomatic segmentation

Verykokou, Ioannidis et al., 2022 [32]	Greece	Segmentation and 3D modelling workflows using dental CBCT data that belong to a patient with periodontitis are evaluated.	NA	NA	3D Slicer		CBCT	The automatic “Grow from seeds” segmentation method.

Janssen, Schreurs et al., 2017 [35]	Netherlands	Segment the grafted alveolar cleft bone.	93%	11	Matlab	NA	CBCT	Semi‐automatic segmentation protocol was used (region growing)

Li, Qiao et al., 2019 [34]	China	Segment the grafted bone.	NA	36	Medraw	16:20	CBCT	Morphological contour interpolation (MCI) algorithm

Abbreviations: AI, artificial intelligence; CBCT, cone‐beam computed tomography; CCN, convolutional neural network; MCI, morphological contour interpolation; PDL, periodontal ligament; RCNN, rapid convolutional neural network.

**Table 4:** Description of the studies (manual segmentation).

**Authors, year of publication**	**Country**	**Aim**	**Evaluation metrics**	**Samples**	**Software**	**Dataset for validation test** **Training:validation:testing**	**Image modality**	**Segmentation methods/tools**
Manavella, Romano et al., 2017 [33]	Italy	Validate a novel procedure for the segmentation of extraction sockets.		9	ImageJ Mimics.		CBCT	Region Growing Segmentation algorithm

Stoop, Chatzivasileiou et al., 2019 [40]	Netherlands	Evaluate the marginal and internal fit of 3D printed resin grafts as they could be used for alveolar ridge augmentation.		6	Amira (FEI Corporate, Oregon, USA)		CBCT	By changing the threshold range.

Gan, Xia et al., 2017 [20]	China	Level set‐based method to segment both tooth and alveolar bone contours slice‐by‐slice from CT images.	95.64%	NA			CBCT	Level set method

Abbreviations: AI, artificial intelligence; CBCT, cone‐beam computed tomography; CCN, convolutional neural network; IAN, inferior alveolar nerve; MCI, morphological contour interpolation; NA, not available; PDL, periodontal ligament; RCNN, rapid convolutional neural network.

**Table 5:** Description of the studies (other than manual segmentation).

**Authors, Year of publication**	**Country**	**Aim**	**Evaluation metrics**	**Samples**	**Image Modality**	**Segmentation Methods/Tools**
Lloréns, Naranjo et al., 2012 [37]	Spain	Segmentation and reconstruction of tissues of the human jaw.	84 ± 1.9	9000 cross‐section (20 patients)	CBCT	Mathematical morphology and on the fuzzy connectedness object extraction.

Kainmueller, Lamecker et al., 2009 [26]	Germany	Automatic segmentation of Mandibular Nerve and Bone from Cone‐Beam CT Data.		106 datasets	CBCT	Statistical Shape Model

Abbreviations: AI, artificial intelligence; CBCT, cone‐beam computed tomography; CCN, convolutional neural network; IAN, inferior alveolar nerve; MCI, morphological contour interpolation; PDL, periodontal ligament; RCNN, rapid convolutional neural network.

### 3.4. Dice coefficient (DC)

Dice coefficient is a metric to compare the predicted segmentation from your AI model with the ground truth segmentation or manual annotation by an expert. The Dice coefficient index was the most common metric used to assess segmentation accuracy, 16 out of 23 studies used this metric to measure the accuracy of the segmentation result. Deep learning methods, particularly the U‐Net architecture, demonstrated superior accuracy in segmenting alveolar bones, with DC ranging from 76% to 98%. Among the 23 studies reviewed, the maximum sample size was 4215, while the minimum was 6, and most of them are at a lower range, except one study. Only four studies focused on segmenting defective alveolar bone, with an average DC score below 90%. Previously, Muller et al. mentioned that a DC score above 90% considered an excellent performance and a DC score between 80% and 90% is considered an acceptable performance [47]. Most of the studies with healthy alveolar bone revealed an excellent performance (avg DC score more than 90%), whereas the study with the defective alveolar bone showed an acceptable performance in comparison with the healthy alveolar bone (avg DC score more than 80%).

### 3.5. Risk of Bias

**Figure 4:**
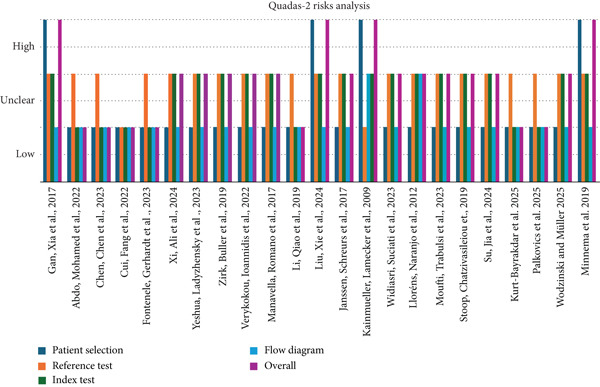
QUADAS‐2 risk analysis.

The results of the risk of bias assessment, conducted using the QUADAS‐2 guidelines (Figure 4), are presented in Table 6. Of the 23 selected studies, 12 were assessed as having an unclear risk of bias [30–33, 35, 37–42, 45]. Out of the remaining studies, seven were deemed low risk [21, 27–29, 34, 43, 44] and four were considered high risk of bias [20, 26, 36, 46].

**Table 6:** QUADAS‐2 checklist.

**Studies**	**Patient selection**	**Reference test**	**Index test**	**Flow diagram**	**Overall**
Gan, Xia et al., 2017 [20]	3	2	2	1	3
Abdo, Mohamed et al., 2022 [27]	1	2	1	1	1
Chen, Chen et al., 2023 [28]	1	2	1	1	1
Cui, Fang et al., 2022 [21]	1	1	1	1	1
Fontenele, Gerhardt et al., 2023 [29]	1	2	1	1	1
Xi, Ali et al., 2024 [42]	1	2	2	1	2
Yeshua, Ladyzhensky et al., 2023 [30]	1	2	2	1	2
Zirk, Buller et al., 2019 [31]	1	2	2	1	2
Verykokou, Ioannidis et al., 2022 [32]	1	2	2	1	2
Manavella, Romano et al., 2017 [33]	1	2	2	1	2
Li, Qiao et al., 2019 [34]	1	2	1	1	1
Liu, Xie et al., 2024 [36]	3	2	2	1	3
Janssen, Schreurs et al., 2017 [35]	1	2	2	1	2
Kainmueller, Lamecker et al., 2009 [26]	3	1	2	2	3
Widiasri, Suciati et al., 2023 [38]	1	2	2	1	2
Lloréns, Naranjo et al., 2012 [37]	1	2	2	2	2
Moufti, Trabulsi et al., 2023 [39]	1	2	2	1	2
Stoop, Chatzivasileiou et al., 2019 [40]	1	2	2	1	2
Su, Jia et al., 2024 [41]	1	2	2	1	2
Kurt‐Bayrakdar et al. 2025 [44]	1	2	1	1	1
Palkovics et al. 2025 [43]	1	2	1	1	1
Wodzinski and Müller 2025 [45]	1	2	2	1	2
Minnema et al. 2019 [46]	3	2	2	1	3

*Note:* High risk = 3. Unclear risk = 2. Low risk = 1.

Furthermore, only a few studies were classified as having an uncertain or high risk of bias in the domain of patient selection, as the CBCT data were not always selected randomly or consecutively [20, 27]. In some cases, the sample selection criteria were not clearly defined. Therefore, the impact of high‐resolution CBCT images on alveolar bone segmentation remains unclear. Additionally, one study [42] used micro‐CT images, which are not widely used in dentistry, to segment the alveolar bone of mice. The applicability of this study is questionable due to patient selection and data acquisition techniques.

## 4. Discussion

This systematic review analyzed the outcomes of different approaches to segmenting CBCT images of alveolar bone to facilitate 3D model creation for periodontal tissue scaffold manufacturing. Studies were categorized into four main groups: deep learning or machine learning techniques, semi‐automated segmentation, manual or threshold‐based segmentation, and alternative segmentation methods.

### 4.1. Challenge in CBCT Image Segmentation

Segmenting alveolar bone from CBCT images is challenging due to its complex anatomy, blurred edges, and homogeneous grayscale values. Interproximal areas are difficult to segment due to similar intensities between adjacent teeth. Segmenting defective alveolar bone in periodontal patients is more challenging than normal bone, as the bone edges are often irregular. Accurate segmentation is crucial for treatment outcomes, but metal artifacts can further complicate the process, especially in periodontal patients. Minnema et al. segmented the CBCT images with metal artifact. The MS‐D network was able to accurately segment bony structures in CBCT scans affected by metal artifacts. [46].

### 4.2. Manual and Semi‐Automated Segmentation

Manual segmentation through adjusting the threshold is reported by three studies for segmenting the alveolar bone [20, 33, 40]. The resorbed alveolar socket was segmented from CBCT images using both region‐growing and automated segmentation techniques in Materialise Mimics software (MiMiC v3.1.0 released by MYNAH Technologies). Segmenting alveolar bone in periodontal patients can be challenging due to irregular edges and heterogeneous intensity. Thresholding approaches are time‐consuming and operator‐dependent. Automated segmentation of the resorbed alveolar socket demonstrated superior results compared to manual segmentation. On the other hand, a few studies mentioned that the automated segmented study revealed a lower output in comparison with the manual segmentation, and further tuning of the model is needed for segmenting the maxillary bone segmentation [29]. Although the segmentation accuracy of deep learning studies for healthy alveolar bone was quite high in most studies, the relative segmentation process was relatively faster than the manual segmentation process [48]. It takes a long time to segment each image by manual thresholding, which is not an ideal process for real clinical practice.

Few studies have developed a semi‐automatic segmentation method for segmenting the alveolar bone from CBCT data [31, 32, 34, 35]. Li et al. segmented the alveolar bone using the morphological contour interpolation (MCI) algorithm, a semi‐automatic segmentation method. However, this method is operator‐dependent, with several steps relying on the operator’s skill. By excluding slices affected by metal artifacts, the study highlighted the method’s limitation in segmenting images with such artifacts. Given the prevalence of dental restorations in dentistry, which can produce artifacts in CBCT images [49, 50], this limitation is significant.

### 4.3. Deep Learning Segmentation

Automated segmentation is the most advanced method for segmenting alveolar bone from CBCT images. Several studies have implemented various types of automated segmentation methods for this purpose. Most studies have demonstrated promising results in segmenting alveolar bone from CBCT data, with U‐Net emerging as the most widely used deep learning method for 3D segmentation of bone [21, 27–30, 36, 38–41]. Deep learning approaches are more flexible and powerful compared to manual and semi‐automatic segmentation methods. The popularity of deep learning methods is due to their higher accuracy and reduced processing time. Deep learning methods can perform segmentation 500 times faster than other methods [21].

Cui et al. segmented alveolar bone and teeth in a large dataset of 4215 patients (4938 CBCT scans) from 15 different centers using a novel AI‐driven segmentation technique [21]. This study included a diverse range of samples, including those with metal artifacts, missing teeth, and restorations, to enhance clinical applicability. The results demonstrated that metal artifacts can affect the segmentation of alveolar bone and teeth from CBCT images. The AI‐driven approach achieved segmentation accuracy comparable to that of expert radiologists. While the segmentation accuracy for defective alveolar bone was 83.5% [30]. More precise training is needed according to the variation of periodontal bone defects for the development of 3D‐printed periodontal scaffolds for periodontal bone defects.

**Figure 5:**
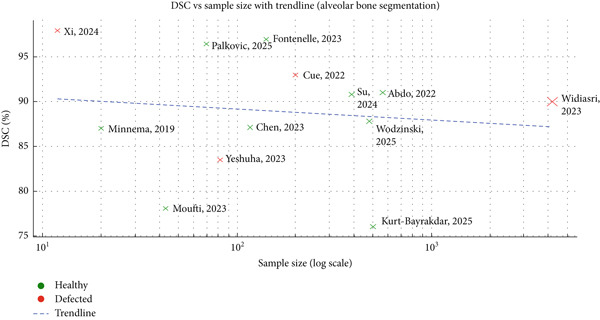
Plot analysis of bone type with sample size and DC score.

Moreover, deep learning methods are less operator‐dependent, and their performance is less susceptible to inter‐observer variability. All included studies proposing deep learning methods based on variations of CNNs have demonstrated promising results in terms of DC. The lowest and highest DC for automatic segmentation methods were 83.5% [30] and 95.4% [29], respectively. In one study, the DC for mandibular bone reported a slightly higher score than the maxillary bone segmentation [21]. The maxillary bone exhibits lower intensity and thinner cortical bone compared to the mandibular bone. Additionally, the heterogeneous intensity of the hard palate poses challenges for segmentation. A major limitation of deep learning methods is the requirement for large datasets to train the algorithm. Inadequate training can hinder the algorithm’s ability to segment anatomical variations. The training process involves selecting a suitable deep learning architecture. A loss function, such as cross‐entropy loss or Dice loss, is employed to quantify the discrepancy between the model’s predictions and ground truth annotations. The model’s parameters are optimized using an algorithm like stochastic gradient descent (SGD) or its variants [51]. The model is then trained on an annotated dataset, iteratively adjusting its parameters to minimize the loss function. A validation set is used to monitor performance and prevent overfitting, while a separate test set assesses the model’s generalization ability [52]. However, DC varies with sample size and bone type. Smaller studies tend to cluster at excellent DC scores, while larger datasets have more moderate DC scores. Healthy alveolar bone revealed a higher DC (average DC score more than 90%) score in comparison with the defective alveolar bone (average DC score below 90%) (Figure 5) [47].

### 4.4. Different Deep Learning Architectures for Segmenting Alveolar Bone

Various types of U‐Net (SegResNet, Attention U‐Net, Mask RCNN, etc.) were implemented to segment the alveolar bone from CBCT images. The segmentation output was quite satisfactory for the U‐Net technique where the DC score ranging from 76% to 98%. However, with a larger sample size and defective alveolar bone, the overall DC score was decreasing in terms of the U‐Net technique T [44]. The two‐stage V‐Net segmentation technique shows promising results in terms of a large sample size, but there is a question regarding the defective alveolar bone [21]. Other than the U‐Net technique ToothFairy2 segmentation architecture showed some satisfactory output for segmenting the mandibular alveolar bone, but the accuracy was lower for segmenting the maxillary bone [45].

### 4.5. Segmentation of Healthy Alveolar Bone vs. Periodontally Defective Alveolar Bone

The studies mentioned above primarily focused on segmenting healthy alveolar bone. Only four studies focused on segmenting alveolar bone defects [32, 42–44]. Xi et al. employed micro‐CT imaging to segment the alveolar bone in mice with periodontitis, developing a reliable, rapid, and precise deep learning‐based automatic segmentation model [42]. Although micro‐CT images offer higher resolution than CBCT images, their limited clinical application in dentistry remains a challenge. To date, only a few studies have applied deep learning segmentation methods specifically to alveolar bone loss due to periodontitis, which revealed poor accuracy and specificity. Palkovics et al. used the SegRes model to segment the alveolar bone in stage III and IV periodontal patients [43]. Although the average dice score was higher, the study used only 57 samples to train the model. On the other hand, Kurt‐Bayrakdar et al. segment the alveolar bone loss due to periodontitis; the overall Dice score (76%) and specificity were quite poor in terms of a deep learning model [44]. Most of the previous studies revealed higher accuracy for segmenting the defective alveolar bone, although the maximum sample size was 82 samples. Only one study revealed low accuracy in comparison with the other studies, but the sample size of that study was 502. This clearly shows that further tuning of the model for segmenting the periodontally affected alveolar bone is needed with a higher sample size. Major limitations included the lack of information on sample size and selection criteria in many studies. Additionally, details regarding CBCT scanning protocols and image resolution were often unclear.

### 4.6. Limitations and Future Directions

Most studies utilized limited training data, hindering the validation of segmentation methods for various anatomical and pathological variations of alveolar bone. Moreover, details of demographic factors, clinical characteristics, and geographic location were not available for all included studies. Most reported models have been developed in controlled research environments, and their integration into real‐world clinical workflows requires validation across larger, multi‐center datasets with heterogeneous imaging conditions. To address this limitation, future research should prioritize training deep learning models on diverse datasets encompassing different types of periodontal defects. Although the U‐Net was found to be the most widely applied architecture due to its strong track record in biomedical segmentation, studies identified in this review demonstrated that its performance varied across different contexts and cohorts. This limitation highlights the need for extensions to the U‐Net and the investigation of novel segmentation architectures towards improved model robustness.

This review highlights the overwhelming methodological shift toward deep learning (DL) techniques, specifically the U‐Net architecture, for alveolar bone segmentation, noting its superior performance over manual and semi‐automated methods. While DL demonstrated high accuracy (DC = 76*%* − 98*%*), particularly for healthy bone, the overall evidence base is critically limited by methodological quality, with a predominant number of studies (approx. 52%) classified with an unclear risk of bias (RoB) due to poor reporting of patient selection criteria. A significant disparity exists in segmentation accuracy between healthy and defective alveolar bone, underscoring a key limitation: Model performance diminishes when exposed to the high clinical variability inherent in large‐scale datasets, encompassing differences in bone density, defect severity, pathology, artifacts, and scanner types. Consequently, while DL holds considerable promise, its clinical applicability for reliable treatment and planning remains questionable. Therefore, future investigation must prioritize refining DL algorithms using large, well‐annotated datasets specifically derived from periodontally compromised patients to ensure accurate bone loss segmentation. Future research should therefore combine architectural innovation with training on diverse, clinically representative datasets to advance the accuracy and reproducibility of periodontal bone segmentation and its applications, including the development of 3D‐printed scaffolds tailored to specific periodontal bone loss conditions.

## 5. Conclusions

• A significant difference of 10% was observed between average DC scores in the segmentation of healthy and defective alveolar bone, which indicates the necessity of further research to refine and apply deep learning methods to DICOM images.
• Segmentation accuracy varies significantly with sample size and bone type. Smaller studies tend to cluster at higher accuracy, while larger datasets reveal lower accuracy and sensitivity. Does this raise questions about how to apply these approaches to population‐level datasets?
• Large‐scale datasets introduce variability in bone density, defect severity, pathology, imaging artifacts, and scanner differences. While this reduces apparent segmentation accuracy, it may also improve model generalizability.
• Deep learning models to date have exhibited varied performance in different contexts, with DC ranging from 76% to 98, indicating context‐dependent performance and the need for continued development of novel deep learning algorithms capable of reliably and precisely segmenting periodontal bone loss across diverse populations.
• Continued improvement and validation of deep learning models are required to support clinical applications, including patient‐tailored 3D‐printed periodontal scaffolding.

## Conflicts of Interest

The authors declare no conflicts of interest.

## Author Contributions

MM, EJ, TFM, MR, and SB contributed to the design of the review. MM developed the search strategy, performed the literature search, synthesis, and interpretations. MM and EJ acted as the primary and secondary reviewer for screening and data extraction, respectively. MR, SB, and TFM acted as the primary and secondary critical appraiser for quality assessment, respectively. All authors contributed to editing and approval of the final manuscript.

## Funding

This study is supported by the James Cook University (10.13039/501100001792) and the International Research Training Program Stipend Scholarship (IRTPRS).

## Data Availability

The data that support the findings of this study are available from the corresponding author upon reasonable request.

## References

[1] Alamri H. M. , Sadrameli M. , Alshalhoob M. A. , and Alshehri M. A. , Applications of CBCT in Dental Practice: A Review of the Literature, General Dentistry. (2012) 60, no. 5, 390–400, 23032226. 23032226

[2] Alrashed S. , Dutra V. , Chu T. M. G. , Yang C. C. , and Lin W. S. , Influence of Exposure Protocol, Voxel Size, and Artifact Removal Algorithm on the Trueness of Segmentation Utilizing an Artificial-Intelligence-Based System, Journal of Prosthodontics-Implant Esthetic and Reconstructive Dentistry. (2024) 33, no. 6, 574–583, 10.1111/jopr.13827. 38305665

[3] Pauwels R. , Araki K. , Siewerdsen J. , and Thongvigitmanee S. S. , Technical Aspects of Dental CBCT: State of the Art, Dentomaxillofacial Radiology. (2015) 44, no. 1, 20140224, 10.1259/dmfr.20140224, 2-s2.0-84919754418, 25263643. 25263643 PMC4277439

[4] Papapanou P. N. , Sanz M. , Buduneli N. , Dietrich T. , Feres M. , Fine D. H. , Flemmig T. F. , Garcia R. , Giannobile W. V. , Graziani F. , Greenwell H. , Herrera D. , Kao R. T. , Kebschull M. , Kinane D. F. , Kirkwood K. L. , Kocher T. , Kornman K. S. , Kumar P. S. , Loos B. G. , Machtei E. , Meng H. , Mombelli A. , Needleman I. , Offenbacher S. , Seymour G. J. , Teles R. , and Tonetti M. S. , Periodontitis: Consensus Report of Workgroup 2 of the 2017 World Workshop on the Classification of Periodontal and Peri-Implant Diseases and Conditions, Journal of Periodontology. (2018) 89, no. Supplement 1, S173–S182, 10.1002/JPER.17-0721, 2-s2.0-85072066206. 29926951

[5] Aimetti M. , Stasikelyte M. , Mariani G. M. , Cricenti L. , Baima G. , and Romano F. , The Flapless Approach With and Without Enamel Matrix Derivatives for the Treatment of Intrabony Defects: A Randomized Controlled Clinical Trial, Journal of Clinical Periodontology. (2024) 51, no. 9, 1112–1121, 10.1111/jcpe.14028, 38859627. 38859627

[6] Ivanovski S. , Vaquette C. , Gronthos S. , Hutmacher D. , and Bartold P. , Multiphasic Scaffolds for Periodontal Tissue Engineering, Journal of Dental Research. (2014) 93, no. 12, 1212–1221, 10.1177/0022034514544301, 2-s2.0-84911907516, 25139362. 25139362 PMC4462800

[7] Verykokou S. , Ioannidis C. , and Angelopoulos C. , CBCT-Based Design of Patient-Specific 3D Bone Grafts for Periodontal Regeneration, Journal of Clinical Medicine. (2023) 12, no. 15, 10.3390/jcm12155023, 37568425. PMC10419991 37568425

[8] Su S. , Liu Y. M. , Zhan L. P. , Gao S. Y. , He C. , Zhang Q. , and Huang X. F. , Evaluation of the Accuracy of Cone-Beam Computed Tomography Image Segmentation of Isolated Tooth Roots Based on the Dynamic Threshold Method, BMC Oral Health. (2023) 23, no. 1, 10.1186/s12903-023-03423-y, 37833773. PMC10571368 37833773

[9] Palkovics D. , Molnar B. , Pinter C. , Gera I. , and Windisch P. , Utilizing a Novel Radiographic Image Segmentation Method for the Assessment of Periodontal Healing Following Regenerative Surgical Treatment, Quintessence International. (2022) 53, no. 6, 492–501, 10.3290/j.qi.b2793209. 35274512

[10] Scarfe W. C. , Azevedo B. , Pinheiro L. R. , Priaminiarti M. , and Sales M. A. , The Emerging Role of Maxillofacial Radiology in the Diagnosis and Management of Patients With Complex Periodontitis, Periodontology 2000. (2017) 74, no. 1, 116–139, 10.1111/prd.12193, 2-s2.0-85018790439. 28429477

[11] Sepehrian M. , Deylami A. M. , and Zoroofi R. A. , Individual Teeth Segmentation in CBCT and MSCT Dental Images Using Watershed, Proceedings of the 2013 20th Iranian Conference on Biomedical Engineering (ICBME), 2013, IEEE, 27–30, 10.1109/ICBME.2013.6782187, 2-s2.0-84899419081.

[12] Tan M. , Cui Z. , Zhong T. , Fang Y. , Zhang Y. , and Shen D. , A Progressive Framework for Tooth and Substructure Segmentation From Cone-Beam CT Images, Computers in Biology and Medicine. (2024) 169, 107839, 10.1016/j.compbiomed.2023.107839, 38150887. 38150887

[13] Hu F. , Chen Z. , and Wu F. , A Novel Difficult-to-Segment Samples Focusing Network for Oral CBCT Image Segmentation, Scientific Reports. (2024) 14, no. 1, 10.1038/s41598-024-55522-7, 38429362. PMC10907706 38429362

[14] Yamashita R. , Nishio M. , Do R. K. G. , and Togashi K. , Convolutional Neural Networks: An Overview and Application in Radiology, Insights Into Imaging. (2018) 9, no. 4, 611–629, 10.1007/s13244-018-0639-9, 2-s2.0-85052299105, 29934920. 29934920 PMC6108980

[15] Lin X. , Xin W. , Huang J. , Jing Y. , Liu P. , Han J. , and Ji J. , Accurate Mandibular Canal Segmentation of Dental CBCT Using a Two-Stage 3D-UNet Based Segmentation Framework, BMC Oral Health. (2023) 23, no. 1, 10.1186/s12903-023-03279-2, 37563606. PMC10416403 37563606

[16] Deeley M. , Chen A. , Datteri R. , Noble J. , Cmelak A. , Donnelly E. , Malcolm A. , Moretti L. , Jaboin J. , Niermann K. , Yang E. S. , Yu D. S. , Yei F. , Koyama T. , Ding G. X. , and Dawant B. M. , Comparison of Manual and Automatic Segmentation Methods for Brain Structures in the Presence of Space-Occupying Lesions: A Multi-Expert Study, Physics in Medicine & Biology. (2011) 56, no. 14, 10.1088/0031-9155/56/14/021, 2-s2.0-79960349087, 21725140. PMC3153124 21725140

[17] Gong Y. H. , Zhang J. , Cheng J. , Yuan W. , and He L. , Automatic Tooth Segmentation for Patients With Alveolar Clefts Guided by Tooth Descriptors, Biomedical Signal Processing and Control. (2024) 90, 105821, 10.1016/j.bspc.2023.105821.

[18] Cui Z. , Li C. , and Wang W. , ToothNet: Automatic Tooth Instance Segmentation and Identification From Cone Beam CT Images, Proceedings of the IEEE/CVF Conference on Computer Vision and Pattern Recognition, 2019, IEEE, 6368–6377.

[19] Radon J. , 1.1 über die bestimmung von funktionen durch ihre integralwerte längs gewisser mannigfaltigkeiten, Classic Papers in Modern Diagnostic Radiology. (2005) 5, no. 21.

[20] Gan Y. , Xia Z. , Xiong J. , Li G. , and Zhao Q. , Tooth and Alveolar Bone Segmentation From Dental Computed Tomography Images, IEEE Journal of Biomedical and Health Informatics. (2017) 22, no. 1, 196–204, 10.1109/JBHI.2017.2709406, 2-s2.0-85040311395, 28574371. 28574371

[21] Cui Z. , Fang Y. , Mei L. , Zhang B. , Yu B. , Liu J. , Jiang C. , Sun Y. , Ma L. , Huang J. , Liu Y. , Zhao Y. , Lian C. , Ding Z. , Zhu M. , and Shen D. , A Fully Automatic AI System for Tooth and Alveolar Bone Segmentation From Cone-Beam CT Images, Nature Communications. (2022) 13, no. 1, 10.1038/s41467-022-29637-2, 35440592. PMC9018763 35440592

[22] Minnema J. , Wolff J. , Koivisto J. , Lucka F. , Batenburg K. J. , Forouzanfar T. , and van Eijnatten M. , Comparison of Convolutional Neural Network Training Strategies for Cone-Beam CT Image Segmentation, Computer Methods and Programs in Biomedicine. (2021) 207, 106192, 10.1016/j.cmpb.2021.106192, 34062493. 34062493

[23] Moher D. , Liberati A. , Tetzlaff J. , Altman D. G. , and PRISMA Group , Preferred Reporting Items for Systematic Reviews and Meta-Analyses: The PRISMA Statement, International Journal of Surgery. (2010) 8, no. 5, 336–341, 10.1016/j.ijsu.2010.02.007, 2-s2.0-77955171857. 20171303

[24] Belur J. , Tompson L. , Thornton A. , and Simon M. , Interrater Reliability in Systematic Review Methodology: Exploring Variation in Coder Decision-Making, Sociological Methods & Research. (2021) 50, no. 2, 837–865, 10.1177/0049124118799372, 2-s2.0-85055491530.

[25] Whiting P. F. , Rutjes A. W. , Westwood M. E. , Mallett S. , Deeks J. J. , Reitsma J. B. , Leeflang M. M. , Sterne J. A. , Bossuyt P. M. , and QUADAS-2 Group , QUADAS-2: A Revised Tool for the Quality Assessment of Diagnostic Accuracy Studies, Annals of Internal Medicine. (2011) 155, no. 8, 529–536, 10.7326/0003-4819-155-8-201110180-00009, 2-s2.0-80054740636. 22007046

[26] Kainmueller D. , Lamecker H. , Seim H. , Zinser M. , and Zachow S. , Automatic Extraction of Mandibular Nerve and Bone from Cone-Beam CT Data, Proceedings of the 12th International Conference on Medical Image Computing and Computer-Assisted Intervention (MICCAI2009), 2009, Springer, 76–83, 10.1007/978-3-642-04271-3_10, 2-s2.0-79551685297. 20426098

[27] Abdo Y. , Mohamed N. , Alsawaf M. , and Elsaeed M. , Teeth and Jaw Segmentation From CBCT Images Using 3D Deep Learning Models, Proceedings of the 18th International Computer Engineering Conference, ICENCO 2022, 2022, IEEE, 25–30, 10.1109/ICENCO55801.2022.10032524.

[28] Chen Z. , Chen S. , and Hu F. , CTA-UNet: CNN-Transformer Architecture UNet for Dental CBCT Images Segmentation, Physics in Medicine and Biology. (2023) 68, no. 17, 175042, 10.1088/1361-6560/acf026, 37579767. 37579767

[29] Fontenele R. C. , Gerhardt M. D. , Picoli F. F. , Van Gerven A. , Nomidis S. , Willems H. , Freitas D. Q. , and Jacobs R. , Convolutional Neural Network-Based Automated Maxillary Alveolar Bone Segmentation on Cone-Beam Computed Tomography Images, Clinical Oral Implants Research. (2023) 34, no. 6, 565–574, 10.1111/clr.14063, 36906917. 36906917

[30] Yeshua T. , Ladyzhensky S. , Abu-Nasser A. , Abdalla-Aslan R. , Boharon T. , Itzhak-Pur A. , Alexander A. , Chaurasia A. , Cohen A. , Sosna J. , Leichter I. , and Nadler C. , Deep Learning for Detection and 3D Segmentation of Maxillofacial Bone Lesions in Cone Beam CT, European Radiology. (2023) 33, no. 11, 7507–7518, 10.1007/s00330-023-09726-6, 37191921. 37191921

[31] Zirk M. , Buller J. , Zöller J. E. , Heneweer C. , Kübler N. , and Lentzen M. P. , Volumetric Analysis of MRONJ Lesions by Semiautomatic Segmentation of CBCT Images, Oral and Maxillofacial Surgery. (2019) 23, no. 4, 465–472, 10.1007/s10006-019-00805-x, 31673817. 31673817

[32] Verykokou S. , Ioannidis C. , and Angelopoulos C. , Evaluation of 3D Modeling Workflows Using Dental CBCT Data for Periodontal Regenerative Treatment, Journal of Personalized Medicine. (2022) 12, no. 9, 10.3390/jpm12091355, 36143140. PMC9503221 36143140

[33] Manavella V. , Romano F. , Garrone F. , Terzini M. , Bignardi C. , and Aimetti M. , A Novel Image Processing Technique for 3D Volumetric Analysis of Severely Resorbed Alveolar Sockets With CBCT, Minerva Stomatologica. (2017) 66, no. 3, 81–90, 10.23736/S0026-4970.17.04029-8, 2-s2.0-85019417497, 28181789. 28181789

[34] Li Y. , Qiao S. C. , Gu Y. X. , Zhang X. M. , Shi J. Y. , and Lai H. C. , A Novel Semiautomatic Segmentation Protocol to Evaluate Guided Bone Regeneration Outcomes: A Pilot Randomized, Controlled Clinical Trial, Clinical Oral Implants Research. (2019) 30, no. 4, 344–352, 10.1111/clr.13420, 2-s2.0-85063446838, 30854705. 30854705

[35] Janssen N. G. , Schreurs R. , Bittermann G. K. P. , Borstlap W. A. , Koole R. , Meijer G. J. , and Maal T. J. J. , A Novel Semi-Automatic Segmentation Protocol for Volumetric Assessment of Alveolar Cleft Grafting Procedures, Journal of Cranio-Maxillofacial Surgery. (2017) 45, no. 5, 685–689, 10.1016/j.jcms.2017.02.018, 2-s2.0-85015638154, 28336322. 28336322

[36] Liu Y. , Xie R. , Wang L. F. , Liu H. P. , Liu C. , Zhao Y. M. , Bai S. Z. , and Liu W. Y. , Fully Automatic AI Segmentation of Oral Surgery-Related Tissues Based on Cone Beam Computed Tomography Images, International Journal of Oral Science. (2024) 16, no. 1, 10.1038/s41368-024-00294-z. PMC11079075 38719817

[37] Lloréns R. , Naranjo V. , López F. , and Alcañiz M. , Jaw Tissues Segmentation in Dental 3D CT Images Using Fuzzy-Connectedness and Morphological Processing, Computer Methods and Programs in Biomedicine. (2012) 108, no. 2, 832–843, 10.1016/j.cmpb.2012.05.014, 2-s2.0-84867401288, 22789466. 22789466

[38] Widiasri M. , Suciati N. , Fatichah C. , Astuti E. R. , Putra R. H. , and Arifin A. Z. , Alveolar Bone and Mandibular Canal Segmentation on Cone Beam Computed Tomography Images Using U-Net, Proceedings of the 2023 8th International Conference on Instrumentation, Control, and Automation (ICA), 2023, IEEE, 36–41, 10.1109/ICA58538.2023.10273108.

[39] Moufti M. A. , Trabulsi N. , Ghousheh M. , Fattal T. , Ashira A. , and Danishvar S. , Developing an Artificial Intelligence Solution to Autosegment the Edentulous Mandibular Bone for Implant Planning, European Journal of Dentistry. (2023) 17, no. 4, 1330–1337, 10.1055/s-0043-1764425, 37172946. 37172946 PMC10756774

[40] Stoop C. C. , Chatzivasileiou K. , Berkhout W. E. R. , and Wismeijer D. , Marginal and Internal Fit of 3D Printed Resin Graft Substitutes Mimicking Alveolar Ridge Augmentation: An *In Vitro* Pilot Study, PLoS ONE. (2019) 14, no. 4, e0215092, 10.1371/journal.pone.0215092, 2-s2.0-85064346838, 30986268. 30986268 PMC6464328

[41] Su S. , Jia X. , Zhan L. , Gao S. , Zhang Q. , and Huang X. , Automatic Tooth Periodontal Ligament Segmentation of Cone Beam Computed Tomography Based on Instance Segmentation Network, Heliyon. (2024) 10, no. 2, e24097, 10.1016/j.heliyon.2024.e24097. 38293338 PMC10827460

[42] Xi R. , Ali M. , Zhou Y. , and Tizzano M. , A Reliable Deep-Learning-Based Method for Alveolar Bone Quantification Using a Murine Model of Periodontitis and Micro-Computed Tomography Imaging, Journal of Dentistry. (2024) 146, 105057, 10.1016/j.jdent.2024.105057, 38729290. 38729290 PMC11288397

[43] Palkovics D. , Molnar B. , Pinter C. , García-Mato D. , Diaz-Pinto A. , Windisch P. , and Ramseier C. A. , Automatic Deep Learning Segmentation of Mandibular Periodontal Bone Topography on Cone-Beam Computed Tomography Images, Journal of Dentistry. (2025) 159, 105813, 10.1016/j.jdent.2025.105813. 40373868

[44] Kurt-Bayrakdar S. , Bayrakdar İ. Ş. , Kuran A. , Çelik Ö. , Orhan K. , and Jagtap R. , Advancing Periodontal Diagnosis: Harnessing Advanced Artificial Intelligence for Patterns of Periodontal Bone Loss in cone-beam Computed Tomography, Dento Maxillo Facial Radiology. (2025) 54, no. 4, 268–278, 10.1093/dmfr/twaf011. 39908459 PMC12038236

[45] Wodzinski M. and Müller H. , Automatic Multi-Structure Segmentation in Cone Beam Computed Tomography Volumes Using Deep Encoder-Decoder Architectures, Proceedings of the International Conference on Medical Image Computing and Computer-Assisted Intervention, 2025, Springer, 63–71, 10.1007/978-3-031-88977-6_7.

[46] Minnema J. , van Eijnatten M. , Hendriksen A. A. , Liberton N. , Pelt D. M. , Batenburg K. J. , Forouzanfar T. , and Wolff J. , Segmentation of Dental Cone-Beam CT Scans Affected by Metal Artifacts Using a Mixed-Scale Dense Convolutional Neural Network, Medical Physics. (2019) 46, no. 11, 5027–5035, 10.1002/mp.13793, 2-s2.0-85073793389, 31463937. 31463937 PMC6900023

[47] Müller D. , Soto-Rey I. , and Kramer F. , Towards a Guideline for Evaluation Metrics in Medical Image Segmentation, BMC Research Notes. (2022) 15, no. 1, 10.1186/s13104-022-06096-y, 35725483. PMC9208116 35725483

[48] Cui D. D. , Long Y. , Yan Y. , Li C. , Yang Y. T. , Zhong J. L. , and Yang R. , Three-Dimensional Magnetic Resonance Imaging Fast Field Echo Resembling a Computed Tomography Using Restricted Echo-Spacing Sequence Is Equivalent to 3-Dimensional Computed Tomography in Quantifying Bone Loss and Measuring Shoulder Morphology in Patients With Shoulder Dislocation, Journal of Arthroscopic & Related Surgery. (2024) 40, no. 6, 1777–1788, 10.1016/j.arthro.2023.12.016. 38154531

[49] Mohammed M. , Rahman N. , and Samsudin A. H. Z. , The Impact of Different Types of Orthodontic Appliances and Its Location in Producing CT Scan Artefacts, Sains Malaysiana. (2021) 50, no. 10, 3067–3075, 10.17576/jsm-2021-5010-19.

[50] Mohammed M. , Rahman N. A. , and Samsudin A. H. Z. , Artefact of Fixed Orthodontic Auxiliary Appliance in Craniofacial CT Image, Orthodontic Waves. (2021) 80, no. 1, 41–46, 10.1080/13440241.2021.1891398.

[51] Taye M. M. , Understanding of Machine Learning With Deep Learning: Architectures, Workflow, Applications and Future Directions, Computers. (2023) 12, no. 5, 10.3390/computers12050091.

[52] Ahmed S. F. , Alam M. S. B. , Hassan M. , Rozbu M. R. , Ishtiak T. , Rafa N. , Mofijur M. , Shawkat Ali A. , and Gandomi A. H. , Deep Learning Modelling Techniques: Current Progress, Applications, Advantages, and Challenges, Artificial Intelligence Review. (2023) 56, no. 11, 13521–13617, 10.1007/s10462-023-10466-8.

